# Neuroprotective Effect of Fresh Gac Fruit Parts Against β-Amyloid-Induced Toxicity and Its Influence on Synaptic Gene Expression in HT-22 Cell Model

**DOI:** 10.3390/molecules30244767

**Published:** 2025-12-13

**Authors:** Asif Ali, Chih-Li Lin, Chin-Kun Wang

**Affiliations:** 1 Department of Nutrition, Chung Shan Medical University, 110, Section 1, Jianguo North Road, Taichung 40201, Taiwan; asiffstpk@gmail.com; 2 Institute of Medicine, Chung Shan Medical University, 110, Section 1, Jianguo North Road, Taichung 40201, Taiwan; dll@csmu.edu.tw

**Keywords:** gac fruit, Aβ toxicity, HT-22 cell, neurodegeneration, neuroprotection, synaptic function, PSD95, neurexin, neuroligin

## Abstract

Neurodegenerative diseases (NDs) have emerged as a significant global health crisis, disproportionately affecting the aging population. As longevity increases, the incidence, healthcare costs, and caregiver burden associated with NDs are escalating at an alarming rate. As of recent data, NDs such as Alzheimer’s disease (AD) are not only significant health burdens but also reflect a complex interplay between socio-economic factors and healthcare systems worldwide. Gac fruit (*Momordica cochinchinensis)* is a rich source of bioactive compounds that has been used as food and traditional medicine. Gac fruit ameliorates memory deficits, enhances beta amyloid (Aβ)_1–42_ clearance, and induces neurite outgrowth. In this study, we examined the anti-neurodegenerative and synaptic improvement effect of fresh gac fruit parts extracts (FGPEs) produced from different solvents. Results showed that the 80% ethanol extract of peel (PE-EtOH) and ethyl acetate extract of seed (SE-EtOAc) significantly protected HT-22 cells by attenuating Aβ-induced cell death, intracellular reactive oxygen species (ROS) production, mitochondrial dysfunction, and synaptic dysfunction. PE-EtOH protected synaptic functions by significantly increasing the postsynaptic density protein-95 (PSD-95) and reducing the neurexin 2 mRNA expression. In contrast, SE-EtOAc increased the PSD-95 and neurexin 3 and reduced the neurexin 2 expressions. These findings indicate that PE-EtOH and SE-EtOAc could have great potential in ameliorating Aβ-induced toxicity in an HT-22 cell model.

## 1. Introduction

According to the World Health Organization (WHO, 2024), neurological disorders are the leading cause of ill health and disability worldwide [1]. According to the statistics from Alzheimer’s Disease International, every 3 s a person develops dementia. The number of people living with dementia is expected to reach 70 million in 2030 and 139 million by 2050. AD is the most common form of dementia, contributing to 60–70% of the dementia cases [2]. Age, genetics, stress, and environmental factors can be associated with neurological disorders [3,4]. The elderly population are predominantly at risk of being affected by neurodegeneration as the disease severity accelerates with aging [3]. The onset of Aβ accumulation began at age 60.7–73.1 years; its prevalence continues as age increases. The aggregation of Aβ is the hallmark pathological sign of AD [5]. Aβ peptide is produced through the sequential proteolysis of a transmembrane protein, amyloid precursor protein (APP), by β- and γ-secretases, and it self-aggregates, especially in oligomer form, which increases its toxicity [6]. Aβ induced the neurotoxicity, which causes neuronal cell death. Although several toxic pathways were implicated, it is known that Aβ’s interaction with mitochondria cause the depolarization of the mitochondrial membrane potential and increase the production of ROS, which leads to an increase in oxidative stress that in turn leads to neuronal dysfunction and apoptosis [7]. Furthermore Aβ-induced mitochondrial dysfunction indicated the loss of mitochondrial ability to support synaptic function. At the synapse, mitochondria provide ATP for neurotransmitter packaging, exocytosis of the synaptic vesicles, neurotransmitter reuptake, calcium (Ca^2+^) buffering via the Ca^2+^-ATPase, and modulation of the activity of ion channels, pumps, and receptors [8]. In parallel, the presynaptic and postsynaptic membranes of neurons contain cell adhesion proteins neurexin (Nrxs) and neuroligin (Nlgn). Neurexin 2 and neurexin 3 regulate synapse formation, specify synapse type, and control neurotransmitter release. Neuroligin 2 binds the scaffolding protein gephyrin activate collybistin, a key protein in the inhibitory gamma-amino butyric acid (GABA)-ergic synapse. Complexes of neuroligin 2, gephyrin, and collybistin are sufficient for the cell-autonomous clustering of inhibitory neurotransmitter receptors. Trans-synaptic interactions between Nrxs and Nlgns are essential for synapse mediating signaling across the synapse and shaping the properties of neural networks by specifying synaptic functions [9]. PSD-95 is a major postsynaptic scaffolding protein at excitatory glutamatergic synapses, which anchor a variety of receptors and ion channels, and transmembrane cell adhesion molecules neuroligin 2, neurexin 2, and neurexin 3 for the formation and proper function of synapses. PSD-95 directly interacts with both Tau and N-methyl-D-aspartic acid receptors (NMDAR) to form the NMDAR/PSD-95/Tau/Fyn complex, which regulates synaptic strength, organizes signaling complexes, and supports activity-dependent plasticity [10,11]. Aβ triggers alterations in the expression level and distribution of PSD-95, which in turn affects the level and interaction between neurexin and neuroligin [9]. PSD-95, neurexin, and neuroligin disruption is a crucial part in neurodegenerative disease [9,12]. It indicates the need for therapeutic strategies to reduce the Aβ-induced oxidative stress and synaptic damage and protect against AD [13].

In recent decades, there has been growing focus on identifying plant-derived natural bioactives with neuroprotective potential. Among these are polyphenols that have been known to protect against neurodegeneration due to their capacity to modulate diverse cellular pathways implicated in the disease [14]. Previous studies showed that gac fruit is a rich source of polyphenols, including phenolic, flavonoids, and carotenoids, which contribute to their antioxidant, anti-inflammatory, and immunomodulatory activity and has been used as both food and traditional medicine [15,16,17,18]. Gac fruit (*Momordica cochinchinensis*) is a tropical fruit that belongs to the *Cucurbitaceae* family, native to Southeast Asia, and produces red fruit (mature) in July and is harvested in August [15]. The amount of phenolic compounds varies by fruit maturity stage and the extraction method [19,20]. The phytochemical levels progressively increase during maturation, with the highest levels typically observed in fully ripe fruits with red skin color [18]. Gac fruits with orange patches to fully orange skin with red aril are considered to be in stage four of maturity, which is the ready-to-eat stage and contains the highest level of phytochemicals [20]. Gac fruit size varied with a weight range of 359.17 to 588.33 g, and its parts such as peel, pulp, aril, and seed comprise 16.65 to 20.60%, 34.06 to 41.58%, 15.64 to 18.64%, and 23.11 to 29.70% portion per gac fruit, respectively [21]. The moisture content of gac fruit varies by maturity and the part of the fruit. The stage four matured fresh gac fruit peel, pulp, aril, and seed contained 83.97 ± 0.29, 91.42 ± 0.17, 82.31 ± 0.25, and 30.36 ± 0.56% moisture content, respectively [20]. Gac fruit peel contains many bioactive compounds but is usually discarded as waste. Gac fruit pulp and arils have been used as functional foods and food supplement ingredients [18,22,23,24]. The gac fruit aril is commonly used as a colorant for the red glutinous rice [25]. A previous study showed that adult zebrafish that received gac aril extract significantly increased time spent in the green arm and reduced latency time, indicating the memory improvement effect [26]. The seed part has been used as medicine worldwide [27]. Mazzio et al. (2018) found that a 17 kDa stable protein within the gac fruit seed has the capacity to induce neurite outgrowth [28]. Despite the well-documented health effect of gac fruit parts, limited studies have explored its potential neuroprotective and mechanistic or functional role regarding neuronal survival or synaptic function, particularly against Aβ-induced neuronal damage.

This study is aimed to investigate the neuroprotective potential of fresh gac fruit parts (peel, pulp, aril, and seed) extracts with different polar solvents (water, 80% ethanol, ethyl acetate, and n-hexane) against Aβ-induced neuronal cell damage and synaptic dysfunction using HT-22 cells. We hypothesized that different polar solvents including water, 80% 74 ethanol, ethyl acetate, and n-hexane extracts of fresh gac fruit parts could attenuate Aβ toxicity by reducing oxidative stress, improving mitochondrial function, and modulating the synaptic genes. This research will fill the gap regarding the neuroprotective effect of fresh gac fruit parts and provide the mechanistic insight that is currently missing in the literature.

## 2. Results

### 2.1. Extraction Yield

Fresh gac fruit parts (peel, pulp, aril, and seed) were extracted with different polar solvents, including water, 80% ethanol, ethyl acetate, and n-hexane. Sixteen extracts were obtained including the following: water extract of peel (PE-H_2_O), 80% ethanol extract of peel (PE-EtOH), ethyl acetate extract of peel (PE-EtOAc), n-hexane extract of peel (PE-hexane), water extract of pulp (PU-H_2_O), 80% ethanol extract of pulp (PU-EtOH), ethyl acetate extract of pulp (PU-EtOAc), n-hexane extract of pulp (PU-hexane), water extract of aril (AR-H_2_O), 80% ethanol extract of aril (AR-EtOH), ethyl acetate extract of aril (AR-EtOAc), n-hexane extract of aril (AR-hexane), water extract of seed (SE-H_2_O), 80% ethanol extract of seed (SE-EtOH), ethyl acetate extract of seed (SE-EtOAc), and n-hexane extract of seed (SE-hexane). After drying, the extracted yields (Yield (%) = (weight of dried extract ÷ weight of raw material before extraction) × 100) of the extracts were calculated. The water extracts of peel (2.34 ± 1.42%) and pulp (1.36 ± 0.85%) showed a higher yield than n-hexane extracts of peel (0.06 ± 0.03%) and pulp (0.05 ±0.02%). In contrast, the n-hexane extract of aril (3.25 ± 0.23%) and seed (8.33 ± 1.2%) showed a higher yield than the water extract of aril (1.97 ± 0.73%) and seed (1.42 ± 1.01%). Overall, 80% ethanol extracts of peel (3.33 ± 0.12%), pulp (3.23 ± 0.16%), and aril (10.14 ± 1. 11%) and the ethyl acetate extract of seed (14.45 ± 1.09%) had a significantly higher yield than other extracts. These results indicated that the peel and pulp may contain higher polar solvents favoring compounds, while the aril and seed contain higher non-polar-favoring compounds, which may account for the yield difference.

### 2.2. Total Phenolic, Flavonoids, and Condensed Tannin Contents in FGPE

The Folin–Ciocalteu method was used to analyze total phenolic contents, including both flavonoids and other non-flavonoid compounds that contain phenolic groups. Results showed that the ethyl acetate extracts of peel, pulp, aril, and seed contain higher total phenolic contents (47.81 ± 2.50, 36.72 ± 3.05, 25.44 ± 3.29, and 31.03 ± 9.74 mg GAE/g extract DW, respectively) than the others (Table 1). The flavonoid contents were analyzed by using aluminum chloride methods. The results showed that the ethyl acetate extracts of peel, pulp, aril, and seed contain higher flavonoid contents (16.09 ± 2.81, 13.29 ± 0.34, 23.35 ± 1.08, and 16.85 ± 0.70 mg QE/g extract DWE, respectively) than the others. Further, the total condensed tannins were determined by the acidified vanillin method. The 80% ethanol extracts of peel, pulp, and seed showed higher total condensed tannin content than water, ethyl acetate, and n-hexane extracts. These results indicated that the phenolic contents in ethyl acetate extracts were primarily due to flavonoid compounds, while the phenolic compounds in 80% ethanol extracts were due to both flavonoids and non-flavonoid phenolic compounds. Although the ethyl acetate extracts showed higher phenolic and flavonoid contents, 80% ethanol extracts had lower flavonoids but higher levels of condensed tannins.

### 2.3. Neuroprotective Effect of FGPE Against Aβ-Induced Neurotoxicity

We performed the MTT assay to determine the safe dose of FGPE and memantine (Figure S2) for HT-22 cells. After 24 h of treatment by PU-EtOAc, PU-hexane, and AR-H_2_O, cell viability was significantly reduced at a concentration of 50 µg/mL. PE-hexane, PU-H_2_O, SE-H_2_O, and SE-EtOH significantly reduced cell viability at a concentration of 100 µg/mL. PE-EtOAc and AR-EtOH significantly reduced cell viability at concentrations of 500 µg/mL, respectively. In contrast, PE-H_2_O, PE-EtOH, PU-EtOH, AR-EtOAc, AR-hexane, SE-EtOAc, and SE-hexane did not show toxicity at a concentration of 500 µg/mL (Figure S1). These results demonstrated that the water and 80% ethanol extracts of peel and pulp showed low toxicity, whereas water and 80% ethanol extracts of aril and seed exhibited higher toxicity. In contrast, ethyl acetate and n-hexane extracts of peel and pulp showed higher toxicity, while ethyl acetate and n-hexane extracts of aril and seed exhibited low toxicity.

**Figure 1:**
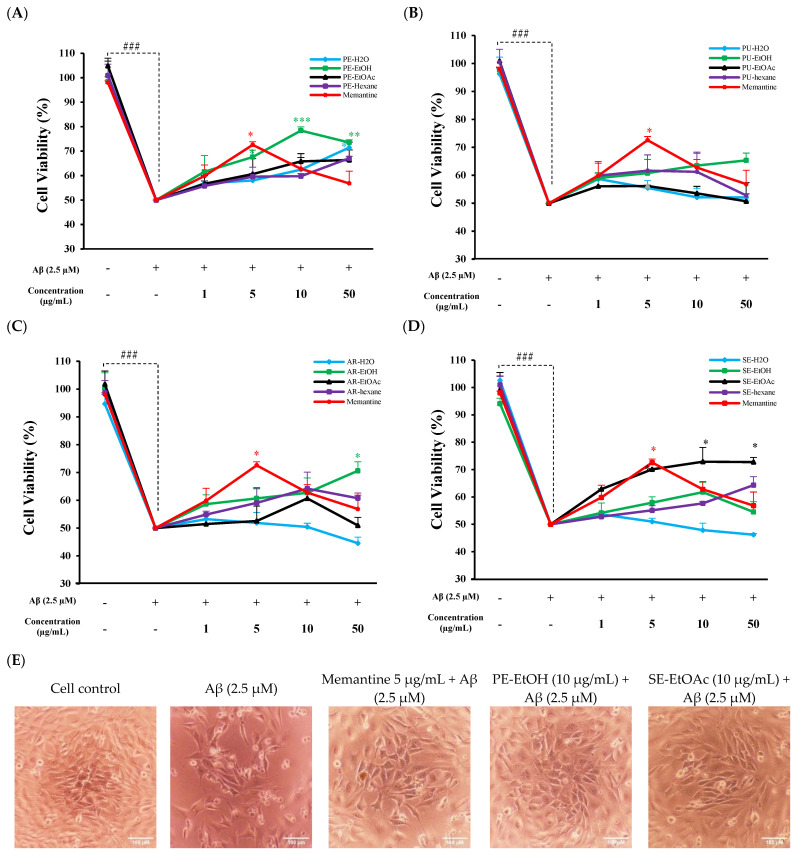
Neuroprotective effect of FGPE and memantine against Aβ-induced toxicity in HT-22. Protective effect of (**A**) peel extracts (PE-H_2_O, PE-EtOH, PE-EtOAc, and PE-hexane) and memantine; (**B**) pulp extracts (PU-H_2_O, PU-EtOH, PU-EtOAc, and PU-hexane) and memantine; (**C**) aril extracts (AR-H_2_O, AR-EtOH, AR-EtOAc, and AR-hexane) and memantine; and (**D**) seed extracts (SE-H_2_O, SE-EtOH, SE-EtOAc, and SE-hexane) and memantine. With (+) and without (−), gac fruit extracts and memantine concentration represented as (1, 5, 10, and 50 µg/mL). (**E**) Cell morphology after PE-EtOH (10 µg/mL), SE-EtOAc (10 µg/mL), or memantine (50 µg/mL) co-treated with Aβ (2.5 µM). Cell viability results were analyzed by comparing control (untreated) cells to Aβ control cells (treated with 2.5 µM Aβ) to assess the toxic effect of Aβ. Additionally, cells co-treated with FGPE or memantine and Aβ were compared to Aβ-treated cells to evaluate the protective effects of FGPE and memantine. PE-H_2_O: water extract of peel; PE-EtOH: 80% ethanol extract of peel; PE-EtOAc: ethyl acetate extract of peel; PE-hexane: n-hexane extract of peel; PU-H_2_O: water extract of pulp; PU-EtOH: 80% ethanol extract of pulp; PU-EtOAc: ethyl acetate extract of pulp; PU-hexane: n-hexane extract of pulp; AR-H_2_O: water extract of aril; AR-EtOH: 80% ethanol extract of aril; AR-EtOAc: ethyl acetate extract of aril; AR-hexane: n-hexane extract of aril; SE-H_2_O: water extract of seed; SE-EtOH: 80% ethanol extract of seed; SE-EtOAc: ethyl acetate extract of seed; and SE-hexane: n-hexane extract of seed. The data are expressed as mean ± standard deviation (SD) (*n* = 3). ^###^ *p* < 0.001 compared with control; * *p* < 0.05, ** *p* < 0.01, and *** *p* < 0.001 compared with Aβ.

The non-toxic concentrations of all extracts were further used to evaluate the protective effects against Aβ-induced cytotoxicity. We treated the HT-22 cell with Aβ_1–42_ for 24 h to induce the neurotoxicity. Results showed that treatment at 2.5 µM Aβ for 24 h reduced the cell viability by 50.00 ± 9.50% and changed the cell morphology, as compared to untreated cells (Figure S3). The co-treatment of 50 µg/mL PE-H_2_O, PE-EtOH, AR-EtOH, and SE-EtOAc significantly protected against Aβ-induced cytotoxicity by increasing the cell viability % (Figure 1A,C,D). Interestingly, 10 µg/mL of PE-EtOH and SE-EtOAc exhibited the highest neuroprotective effect on Aβ-induced cytotoxicity. Whereas PE-EtOAc, PE-hexane, PU-H_2_O, PU-EtOH, PU-EtOAc, PU-hexane, AR-H_2_O, AR-EtOAc, AR-hexane, SE-H_2_O, SE-EtOH, SE-EtOAc, and SE-hexane (1–50 µg/mL) did not protect HT-22 neurotoxicity induced by Aβ_1–42_. In comparison, between PE-EtOH, SE-EtOAc, and memantine, the significant protection *p* value of PE-EtOH, SE-EtOAc, and memantine was 0.001, 0.02, and 0.023, respectively. At 10 µg/mL, PE-EtOH showed significantly higher protection as compared to memantine. Furthermore, PE-EtOH and SE-EtOAc protected HT-22 cell morphology against the Aβ-induced morphology damage, as shown in Figure 1E. These results suggest that both PE-EtOH and SE-EtOAc showed greater neuroprotective effects than other extracts, with PE-EtOH showing a greater effect than memantine. Therefore, PE-EtOH and SE-EtOAc require further investigation to explore their therapeutic potential in neurodegenerative disease.

### 2.4. Aβ-Induced Intracellular Reactive Oxygen Species (ROS) Protected by 80% Ethanol Extract of Peel and Ethyl Acetate Extract of Seed

**Figure 2:**
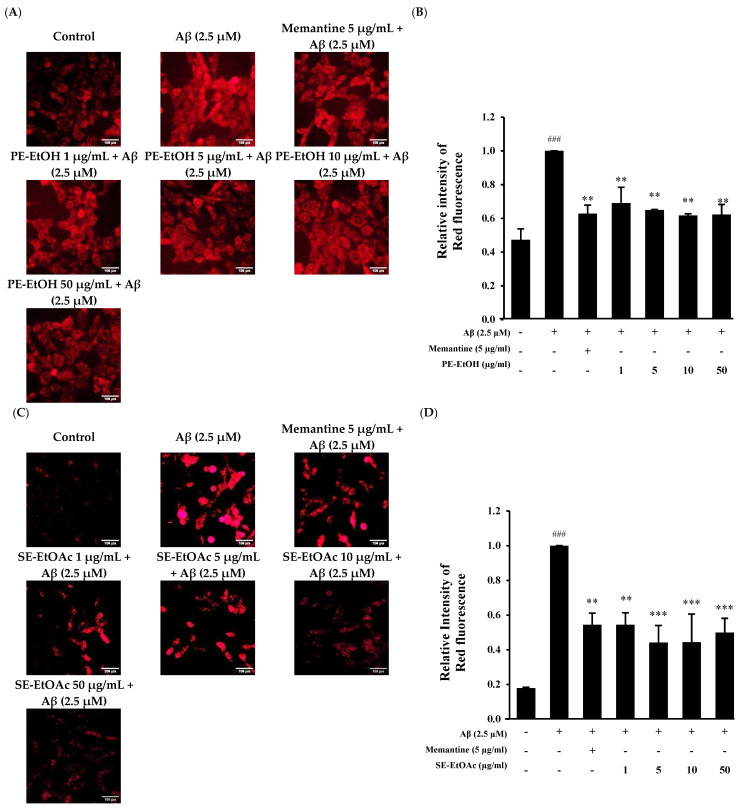
Protective effect of PE-EtOH and SE-EtOAc against Aβ-induced ROS production in HT-22 cells. (**A**) Immunofluorescence images of DHE stained cells after PE-EtOH treatment. Scale bar = 100 µm (**B**) Relative intensity of red fluorescence of PE-EtOH-treated cell. (**C**) Immunofluorescence images of DHE stained cells after SE-EtOAc treatment. Scale bar = 100 µm (**D**) Relative intensity of red fluorescence of SE-EtOAc-treated cell. With (+) and without (−), PE-EtOH and SE-EtOAc concentration represented as (1, 5, 10, and 50 µg/mL). Relative intensity of red fluorescence was calculated by image J software (version 1.54d; National Institutes of Health, Bethesda, MD, USA). DHE: dihydroethidium, PE-EtOH: 80% ethanol extract of peel, and SE-EtOAc: ethyl acetate extract of seed. The data are expressed as mean ± standard deviation (SD) (*n* = 3). ^###^ *p* < 0.001 compared with control, ** *p* < 0.01 and *** *p* < 0.001 compared with Aβ.

Aβ_1–42_ exposure can induce oxidative stress to HT-22 cells by increasing the ROS [29]. In this study, dihydroethidium (DHE) staining was used to detect ROS accumulation in treated cells, and fluorescent microscopy was used to capture images and assess ROS levels. Results showed that 2.5 µM Aβ treatment significantly increased ROS levels as compared control cell. A total of 5 µg/mL memantine with Aβ (2.5 µM)-treated cells suppressed the ROS levels (Figure 2A,C). The co-treatment of PE-EtOH and SE- EtOAc at 1, 5, 10, and 50 µg/mL with 2.5 µM Aβ_1–42_ significantly reduced the intracellular ROS generation in HT-22 cells as compared to the Aβ-treated ones (Figure 2A,C). Quantitative results of ROS accumulation in HT-22 cells show that 2.5 µM Aβ-treated cells exhibit higher red fluorescence as compared with untreated (control) and PE-EtOH, SE-EtOAc, and memantine-treated cells (Figure 2B,D).

This finding suggested that PE-EtOH and SE-EtOAc suppressed ROS production induced by Aβ, similar to that of memantine.

### 2.5. Protective Effect of 80% Ethanol Extract of Peel and Ethyl Acetate Extract of Seed Against Aβ-Induced Mitochondrial Dysfunction

**Figure 3:**
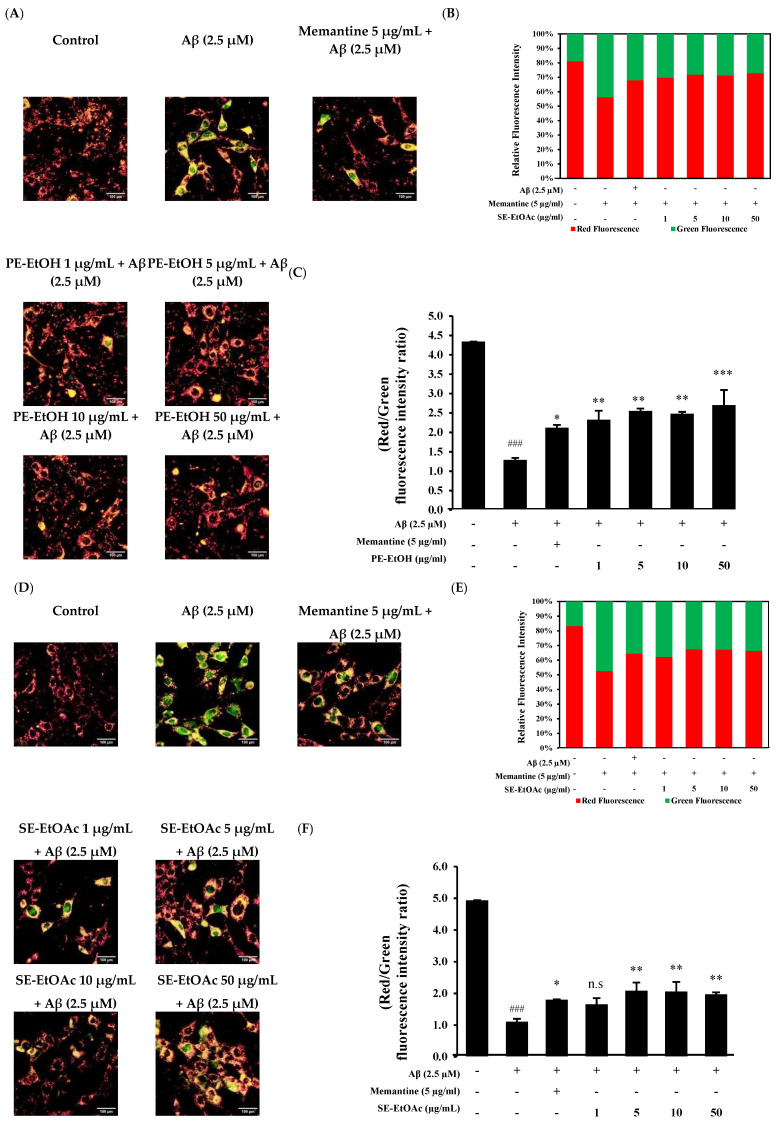
Protective effect of PE-EtOH and SE-EtOAc against Aβ-induced mitochondrial dysfunction in HT-22 cells. (**A**) JC-1-stained cell after PE-EtOH treatment. (**B**) Relative fluorescence intensity of PE-EtOH-treated cell, red fluorescence (healthy mitochondria), and green fluorescence (dysfunctional mitochondria). (**C**) Ratio of red and green fluorescence intensity of PE-EtOH-treated cells. (**D**) JC-1-stained cell after SE-EtOAc treatment. (**E**) Relative fluorescence intensity of red and green fluorescence of SE-EtOAc-treated cell against Aβ. (**F**) Ratio of red and green fluorescence intensity of SE-EtOAc-treated cells. With (+) and without (−), gac PE-EtOH and SE-EtOAc concentration represented as (1, 5, 10, and 50 µg/mL). PE-EtOH: 80% ethanol extract of peel, SE-EtOAc: ethyl acetate extract of seed. The data are expressed as mean ± standard deviation (SD) (*n* = 3). ^###^ *p* < 0.001 compared with control; * *p* < 0.05, ** *p* < 0.01, and *** *p* < 0.001 compared with Aβ; and n.s not significant compared with Aβ.

Aβ-induced mitochondrial dysfunction is known to cause oxidative damage in neurodegeneration [30,31]. The protective effect of PE-EtOH and SE-EtOAc against Aβ-mediated impairment on mitochondrial membrane potential (MMP) was measured. Approximately 2.5 µM Aβ_1–42_ increased the JC-1 monomer green fluorescence intensity and reduced the red fluorescence, indicating a reduced MMP. A total of 5 µg/mL memantine with Aβ (2.5 µM) decreased the green fluorescence and increased the red fluorescence (Figure 3A,D). The co-treatment of PE-EtOH and SE-EtOAc with 2.5 µM Aβ decreased the green fluorescence and restored the Aβ-induced reduction in the red fluorescence/green fluorescence ratio (Figure 3B,E). Quantitative analyses (Figure 3C,F) revealed that PE-EtOH and SE-EtOAc protected against the Aβ-induced decreases in MMP, thereby protecting the Aβ-induced mitochondrial dysfunction. Compared to memantine, both PE-EtOH and SE-EtOAc showed a greater effect on mitochondrial function. The effects of PE-EtOH and SE-EtOAc on ROS production and mitochondrial function were not dose-dependent, as all tested concentrations exhibit similar antioxidant activity and MMP effects.

### 2.6. Effect of 80% Ethanol Extract of Peel and Ethyl Acetate Extract of Seed on Synapse-Related mRNA Expression

**Figure 4:**
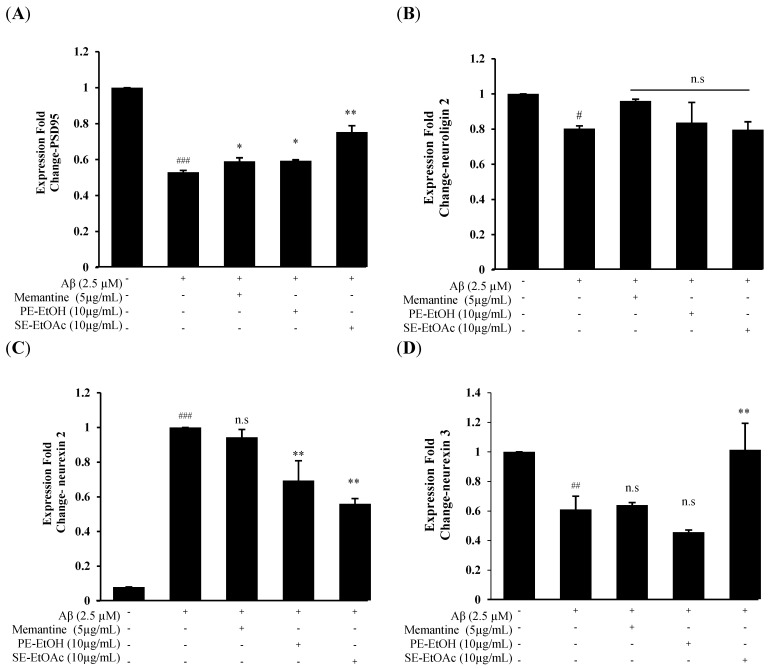
Synaptic-related mRNA expression of PE-ETOH and SE-EtOAc treatment against Aβ-induced synaptic dysfunction. (**A**) PSD95 expression. (**B**) Neuroligin 2 expression. (**C**) Neurexin 2 expression. (**D**) Neurexin 3 expression. With (+) and without (−) PE-EtOH, SE-EtOAc, memantine, and Aβ. PE-EtOH: 80% ethanol extract of peel, SE-EtOAc: ethyl acetate extract of seed. The data are expressed as mean ± standard deviation (SD) (*n* = 3). ^#^ *p* < 0.05, ^##^ *p* < 0.01, and ^###^ *p* < 0.001 compared with control; * *p* < 0.05, ** *p* < 0.01 compared with Aβ; and n.s no significance compared with Aβ.

On the basis of the above finding, PE-EtOH and SE- EtOAc at 10 µg/mL exhibited potential neuroprotection against Aβ among all FGPEs, which were confirmed by DHE and JC-1 staining. Thus, 10 µg/mL of PE-EtOH and SE- EtOAc were used for advanced study on synapse-related mRNA expression, including PSD-95, neuroligin 2, neurexin 2 and neurexin 3. Twenty-four hour treatment of 2.5 µM Aβ significantly downregulated PSD-95, neuroligin 2, and neurexin 3 and upregulated the neurexin 2 expressions compared with the control, indicating the synaptic dysfunction. The co-treatment of 10 µg/mL PE-EtOH and SE-EtOAc with Aβ upregulated PSD-95 and downregulated the neurexin 2 expression (Figure 4A,C). In addition, SE-EtOAc treatment showed the significant upregulation of neurexin 3 expression, which was not seen in PE-EtOH treatment (Figure 4D).

Both PE-EtOH and SE-EtOAc treatment failed to restore the neuroligin 2 expression (Figure 4B). Memantine only upregulated PSD95 expression while it failed to protect neuroligin 2, neurexin 2, and neurexin 3 expression. These results demonstrated that PE-EtOH treatment targeted PSD-95 and neurexin 2, while SE-EtOAc affected a broader category of synaptic proteins to protect the synaptic function.

### 2.7. HPLC Chromatogram of Phenolic Compounds

**Figure 5:**
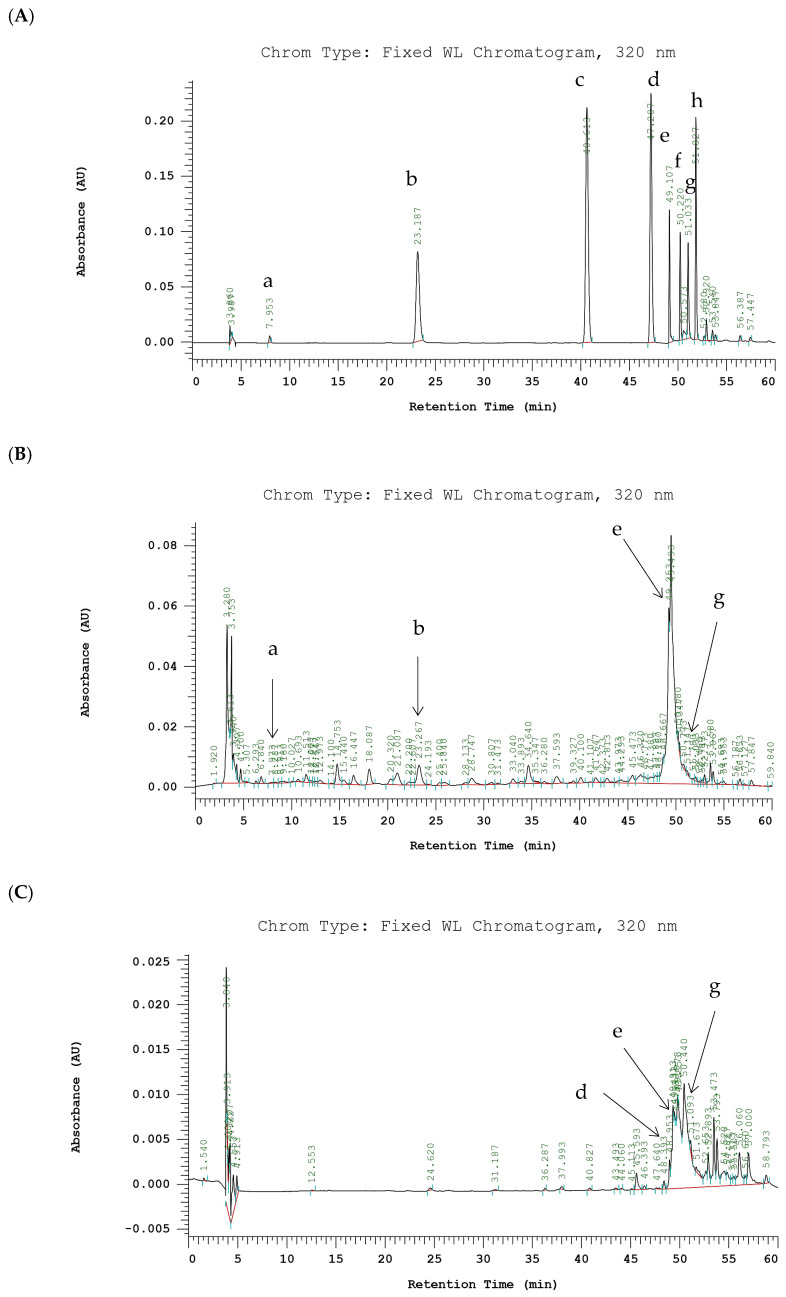
HPLC chromatograms of phenolic compounds of PE-EtOH and SE-EtOAc. (**A**) Standards. (**B**) PE-EtOH. (**C**) SE-EtOAc. Detected at absorbance value 320 nm. (a) Gallic acid, (b) chlorogenic acid, (c) *p*-coumaric acid, (d) sinapic acid, (e) rutin, (f) myricetin, (g) quercetin, and (h) kaempferol. PE-EtOH: 80% ethanol extract of peel, SE-EtOAc: ethyl acetate extract of seed.

Chromatograms of phenolic compounds derived from PE-EtOH and SE-EtOAc are listed in Figure 5.

Previously identified phenolic acids (gallic acid, chlorogenic acid, *p*-coumaric acid, and sinapic acid) and flavonoids (rutin, myricetin, quercetin, and kaempferol) in gac fruit parts were used as a standard to identify the compounds in PE-EtOH and SE-EtOAc [18,32]. In this study, phenolic acids observed in PE-EtOH include gallic acid and chlorogenic acid, while in SE-EtOAc, observed acids include sinapic acid. Chlorogenic acid in PE-EtOH (121.86 ± 24.32 µg/g EDW) was higher than gallic acid, while gallic acid and chlorogenic acid were not observed in SE-EtOAc.

This indicated that PE-EtOH contained more phenolic acids than SE-EtOAc. Furthermore, flavonoid contents including rutin and quercetin were observed in both PE-EtOH and SE-EtOAc (Figure 5). Rutin contents in PE-EtOH (1203.18 ± 15.67) and SE-EtOAc (52.69 ± 10.74) were significantly higher than quercetin (Table 2).

In SE-EtOAc, the flavonoid contents were considerably higher than the phenolic acid. Major peaks in PE-EtOH and SE-EtOAc remain unknown. These results showed that the phenolic compounds of SE-EtOAc were majorly due to flavonoids, and for PE-EtOH, they were due to both phenolic acids and flavonoids.

## 3. Discussion

This study provides nurturing evidence of neuroprotective effects of FGPE against Aβ-induced toxicity in HT-22 cells. We demonstrated the protective effect of FGPE against neuronal damage by preserving cell viability and reducing intracellular ROS production, mitochondrial dysfunction, and synaptic impairment. The results showed that PE-H_2_O, PE-EtOH, AR-EtOH, and SE-EtOAc significantly attenuated Aβ-induced cytotoxicity, as evidenced by enhanced cell viability. PE-EtOH and SE-EtOAc provided significant protection at a concentration of 10 µg/mL, while PE-H_2_O and AR-EtOH required a higher concertation (50 µg/mL) to reach a similar effect (Figure 1A,C,D). These results suggested that PE-EtOH and SE-EtOAc may contain bioactive compounds, which can protect neuronal cells at a lower concentration, a key factor for their potential therapeutic use. Furthermore, PE-EtOH and SE-EtOAc had a higher yield than other extracts of peel and seed. PE-EtOH and SE-EtOAc with a higher protective effect and yield were further investigated. PE-EtOH and SE-EtOAc significantly protected intracellular ROS production and mitochondrial dysfunction. Furthermore, both improved the synaptic function, as evidenced by the regulation of mRNA expression of synaptic proteins including PSD-95, neurexin 2, and neurexin 3 (Figure 4).

The moisture content of gac fruit parts used in this study were similar to the moisture content of maturity-stage-4 gac fruit parts, as previously reported, which contained higher bioactive compounds and oil contents [20]. In this study, ethyl acetate and the ethanol extract of peel, pulp, aril, and seed showed higher total phenolics than water and n-hexane extract (Table 1). These results were in line with a previous study, which reported that the final total phenolic compounds and carotenoids capacity of maturity-stage-4 gac fruit were varied with the extracting solvent polarity (polar or non-polar) [19]. The cytotoxic results of this study demonstrated that the n-hexane extract of peel and pulp and water extracts of aril and seed exhibited higher toxicity in HT-22 cells, which were consistent with previous studies, which demonstrated that gac fruit seed water extract showed considerable cytotoxic effects in normal HaCat, melanoma D24, and C1 cell lines [33]. Water extracts of seed inhibit cell proliferation and induce apoptosis [22,33]. Wimalasiri et al. (2016) showed that the water extract of gac fruit aril significantly reduced cell viability in ARPE-19, MCF-7, and melanoma cells, indicating the toxic nature of water extracts of gac fruit aril [34]. Furthermore, the 80% ethanol extract of seed at a concentration of 500 µg/mL had the most toxic effect toward HT-22 cell viability (13.76 ± 0.13%). In contrast, the 80% ethanol extracts of peel, pulp, and aril at a concentration of 500 µg/mL showed the cell viability of 111.51 ± 2.49%, 113.76 ± 2.22%, and 80.21 ± 1.67%, respectively. These results were in line with a previous study, which showed that the 70% ethanol extract of gac fruit seed at 500 µg/mL was more toxic towards ARPE-19 cells than the 70% ethanol extracts of peel, pulp, and aril [32]. Another study reported that water and ethanol extracts of seed injected intravenously and intramuscularly induced cardiotoxicity and apoptosis, increased ROS production and neutrophil infiltration, and decreased blood flow velocity in zebrafish, which died within several days [22]. This highlights that the water and ethanol extracts induced toxicity through inflammation, oxidative stress, and the apoptosis pathway. Furthermore, water and ethanol extracts of seed contain secondary metabolites such as triterpenoids and saponins. Both in vitro and in vivo studies showed that saponin had potential hepatorenal toxicity. It inhibits the GSK3β/β-catenin pathway, suppresses cell proliferation and adult neurogenesis, and leads to impaired cognitive performance [16].

Aβ is a main component of senile plaque and is toxic to neuronal cells, which induces neuronal cell death [35]. Similarly to a previous study, this study showed that 24 h exposure of 2.5 µM Aβ significantly reduced the cell viability percentage as compared to the untreated cell. Among the FGPEs, only PE-H_2_O, PE-EtOH, AR-EtOH, and SE-EtOAc exhibited protection against Aβ toxicity. Previous evidence has shown that gac oil supplementation enhances the Aβ_1–42_ clearance by upregulating antioxidative enzymes, anti-inflammatory cytokines, and autophagy in rats [36]. The aqueous extract of gac fruit aril reduces the brain acetylcholinesterase (AChE) activity and improves the locomotor functions in zebrafish [26,37]. Aβ-induced intracellular ROS production, mitochondrial dysfunction, and synaptic dysfunction are the hallmarks of cognitive decline and neurodegeneration [38,39]. This finding was in agreement with the results of this study, which showed that Aβ significantly increased the ROS production and reduced the mitochondrial function. Our results showed that PE-EtOH and SE-EtOAc significantly reduced ROS production and improved mitochondrial function, suggesting that their active compounds of PE-EtOH and SE-EtOAc may exert effects by attenuating oxidative stress. The antioxidant property of gac fruit peel and seed was previously confirmed by DPPH, ABTS, and FRAP (ferric-reducing ability of plasma) assay [40]. Further, Ismail et al. (2019) evaluated the antioxidant capacity of gac fruit peel, pulp, aril, and seed; gac fruit peel showed the highest anti-oxidation property, evidence by FRAP [17].

Furthermore, PE-EtOH and SE-EtOAc restored PSD-95 expression in cells exposed to Aβ, suggesting increased synaptic strength and activity. PSD95 is an abundant scaffolding protein in PSD, and it regulates the trafficking and stabilization of glutamate receptors such as α-amino-3-hydroxy-5-methyl-4-isox-azoleproprionic acid receptors (AMPARs) and N-methyl-D-aspartic acid receptors (NMDARs) at the excitatory synapse [41]. Deficiency of PSD95 is associated with disrupted synaptic function, leading to an imbalance between the excitation and inhibition ratio [42]. PSD-95 was bound to the cytoplasmic COOH-termini of synaptic adhesion protein known as neuroligin, which regulates various aspects of excitatory and inhibitory synaptic transmission through trans synaptic bridges with pre-synapse [10]. Neuroligin-2 is a member of neuroligin that acts exclusively at GABAergic inhibitory synapses [43]. Treatment with PE-EtOH, SE-EtOAc, and memantine did not show any significant change in neuroligin-2 expression. Neuroligin-2 is required for scaling up the inhibitor rather than establishing the unitary inhibitory synaptic connections [44]. This indicated that PE-EtOH and SE- EtOAc protected the synaptic function and plasticity by increasing PSD-95 without affecting the neuroligin-2 expression. Moreover, SE-EtOAc treatment downregulated neurexin 2 and normalized neurexin 3 expressions, while PE-EtOH downregulated neurexin 2 without changing neurexin 3 expressions. Neurexin is a presynaptic transmembrane protein, it is encoded by neurexin 1, neurexin 2, and neurexin 3 genes. Alternative splicing at the splice site (SS4) for neurexin 1 and 3 regulates NMDARs and AMPARs, respectively, while the same splicing for neurexin 2 did not regulate either NMDARs and AMPARs, suggesting that neurexin 1 and 3 might promote the synaptic assembly [45,46]. Further deletion of neurexin 2 in the hippocampal region of the brain impairs cognitive flexibility. Thus, neurexin 2 controls the hippocampal synaptic circuit, making it differ from that of neurexin 1, neurexin 3, and other synaptic adhesion molecules [47,48]. Taken together, these results demonstrated that PE-EtOH treatment targeted PSD-95 and neurexin 2, while SE-EtOAc affected a broader category of synaptic proteins to protect the synaptic function. Our finding is in line with the previous finding that gac fruit seed (17 kD protein) can enhance neurite outgrowth, which is closely related to synaptic plasticity [28]. PSD95 and neuroligin are key proteins that regulate neuronal plasticity [49]. Our results suggested that these synaptic proteins might be directly influenced by SE-EtOAc to restore synaptic communication.

Gac fruit is a rich source of bioactive compounds, including carotenoids, phenolic acids, and flavonoids [32]. These bioactive compounds are receiving more attention due to their potential importance for their medicinal properties and wide application in therapeutic and pharmaceutical applications. These active compounds are well-known for their antioxidant properties and critical role in preventing various diseases [50]. Our study results indicated that, in FGPE, flavonoids were the major contributors to the phenolic content of the ethyl acetate extract, while the 80% ethanol extract contained a broader range of phenolics, including both flavonoid and non-flavonoid compounds. PE-EtOAc contained more flavonoids than PE-EtOH, while PE-EtOH exhibited a greater neuroprotective effect than the other peel extracts. SE-EtOAc showed higher phenolic compounds than the other extracts of seed. In addition, PE-EtOH and SE-EtOAc showed a greater protective effect than other extracts, indicating that flavonoids and non-flavonoid phenolic compounds in PE-EtOH and flavonoid compounds in SE-EtOAc contributed to a greater protective effect. The HPLC chromatograms of PE-EtOH and SE-EtOAc were obtained in this study; the known compounds observed in PE-EtOH include gallic acid, chlorogenic acid, rutin, and quercetin, while in SE-EtOAc, the compounds include sinapic acid, rutin, and quercetin (Figure 5). These results were consistent with findings from a previous study, which confirmed the identification of rutin and quercetin in both the peel and seed of gac fruit [32]. Flavonoids can play an important role in neuroprotection, promoting learning, memory, and cognitive function [51]. Rutin has antioxidant properties, as evidenced by the DPPH and free radical scavenging assay [52]. A previous study showed that rutin attenuate Aβ42-induced cytotoxicity in SH-SY5Y neuroblastoma cells by reducing ROS production, improving mitochondrial function, and enhancing the activities of super oxide dismutase, catalase, and glutathione peroxidase. Furthermore, rutin modulates the production of pro-inflammatory cytokines by decreasing TNF-α and IL-1β generation in microglia [53] and enhances the memory in scopolamine-induced memory impairment in zebrafish by enhancing cholinergic neurotransmission [54]. Furthermore, quercetin protected neuronal cell death and reduced synaptic dysfunction by inhibiting the fibril formation and oligomerization of Aβ and activating the NRF2/HO1 pathway in AD models of rodent [55]. The protective action of rutin and quercetin against Aβ42-induced cytotoxicity was found to be of therapeutic value in treating AD [53,56].

Similarly, both PE-EtOH and SE-EtOAc contained significantly higher rutin and showed a greater neuroprotective effect in this study. Further phenolic acids (gallic acid and *p*-coumaric acid) were observed in PE-EtOH. Gallic acid improves the synaptic failure caused by the Aβ peptides [57]. The oral administration of gallic acid disrupted the Aβ_1–42_ aggregation and decreased the neurotoxicity in a 4-month-old APP/PS1 transgenic mouse and reduced its cognitive decline [58]. *p*-coumaric acid protects the chronic restraint stress-induced memory deficit and depression-like behavior by upregulating protein kinase A, phosphorylated cAMP response element binding protein (pCREB), and brain-derived neurotrophic factor (BDNF) expression in chronic restraint stress mice [59]. Sinapic acid observed in SE-EtOAc has antioxidant and anti-inflammatory properties [60]. Sinapic acid reduces the Aβ-induced cell death and cognitive dysfunction by its antioxidative and anti-inflammatory activities. It was reported that sinapic acid protected scopolamine-induced neuroinflammation in mice by inhibiting COX-2 and IL-1β [61]. Previously, the phenolic acids (gallic acid, *p*-hydroxybenzoic acid, chromogenic acid, caffeic acid, *p*-coumaric acid, ferulic acid, cinnamic acid, and sinapic acid), flavonoids (rutin, myricetin, luteolin, quercetin, and apigenin), carotenoids (beta-carotene, lycopene, and lutein), saponin (momordin II), protein, and decanoic acid derivatives, etc., were identified in gac fruit peel, while sinapic acid, quercetin glucoside derivatives, quercetin, oxo-octadecanoic acid, saponin, and protein etc., have been identified in seed [18,28,32]. According to this study, PE-EtOH and SE-EtOAc showed a greater protective effect and improved the synaptic functions more than other extracts, which might be due to rich antioxidants, saponins, carotenoids, phenolic acids, flavonoids, or proteins. However, this study did not identify the specific bioactive compounds predominantly responsible for these effects in gac fruit peel and seed.

To the best of our knowledge, this study was the first study to investigate the effect of FGPE on Aβ-induced neurodegeneration. Our study showed that PE-EtOH and SE-EtOAc protected neuronal cell death, ROS production, and improved mitochondrial function and synaptic function. Our findings suggest that gac fruit peel and seed could have potential therapeutic properties in treating neurodegeneration. However, further study is recommended to explore the specific active compounds and underlying mechanisms of neuroprotection.

## 4. Material and Methods

### 4.1. Chemicals and Reagents

All reagents used in this study were analytical grade and were purchased from Sigma-Aldrich (St. Louis, MO, USA), Merck (Darmstadt, Germany), and Thermo Fisher (Waltham, MA, USA). Double-distilled deionized water was obtained from a Milli-Q purification system (Millipore, Burlington, MA, USA). Fresh gac fruits were obtained from Ideation National Culture and Creativity Ltd. South Dist., Taichung City, Taiwan.

Synthetic powdered Aβ_1–42_ peptide was directly dissolved in anhydrous dimethyl sulfoxide (DMSO) to obtain a 5 mM stock solution. The suspension was briefly vortexed and then subjected to sonication for 10 min to facilitate complete solubilization (Figure S4). The freshly prepared Aβ/DMSO stock was used immediately to minimize spontaneous aggregation and was not applied directly to cells [62]. For experiments, the freshly prepared Aβ_1–42_ stock solution was immediately diluted with pre-warmed DMEM to the final concentrations (100 μM). The final DMSO concentration was kept at DMSO ≤ 0.05% (*v*/*v*) in all treatment and vehicle control groups.

### 4.2. Sample Preparation

Maturity-stage-4 fresh gac fruits were selected and stored at 4 °C before use. Fresh gac fruit was washed with water to remove contamination and cleaned using a sterilized cloth. Fresh gac fruit was weighed and separated into its parts like peel, pulp, aril, and seed. Gac fruit parts percentage and moisture contents were calculated; part percentage and moisture contents varied by the maturity stage [20]. Peel, pulp, aril, and seed were grounded by blender for advanced extraction. All parts were extracted with water, 80% ethanol, ethyl acetate, and n-hexane at a ratio of 1:5 (weight to volume). After grinding each part with solvents for 30 min, the mixture was filtered through 0.45 µm Whatman^®^ qualitative filter paper (Grade 1, Cytiva, Marlborough, MA, USA). Extracts of 80% ethanol, ethyl acetate, and n-hexane were dried using a vacuum rotary evaporator at 50 °C. Water extracts of peel, pulp, aril, and seed were dried using the freeze-drying method under −80 °C. After drying, all extracts were obtained and stored at 4 °C for further use.

### 4.3. Total Phenolic, Flavonoid, and Condensed Tannin Contents

Total phenolic contents were assessed using the Folin–Ciocalteu method with some modifications [18]. Briefly, the 50 µL FGPEs (1 mg/mL) were mixed with 2 mL water and reacted with 1 mL Folin–Ciocalteu solution at room temperature for 5 min with continuous stirring. It was then mixed with 5 mL sodium carbonate (20% (*w*/*v*) in water) for 20 min at room temperature. The absorbance was measured at 735 nm using a UV-visible spectrophotometer (BioTek Instruments, Winooski, VT, USA). The total phenolic content of FGPE was expressed in mg gallic acid equivalent (GAE)/g FGPE dried weight (DW) based on the standard curves of gallic acid solution. Total flavonoid contents were determined by AlCl_3_ colorimetric methods [18]. A total of 100 µL of FGPEs (1 mg/mL) were mixed with 1 mL of methanol and 50 µL AlCl_3_ (5%, (*w*/*v*) in water). After incubation for 30 min at room temperature, absorbance was measured at 225 nm. Total flavonoid content of FGPE was expressed in mg quercetin equivalent (QE)/g FGPE DW. Condensed tannin contents were determined by the acidified vanillin method [62]. Approximately 100 µL of FGPEs (1 mg/mL) were mixed with 600 µL vanillin (4% (*w*/*v*) in methanol) and 300 µL of concentrated HCl. The mixture was incubated for 15 min, and absorbance was measured at 500 nm. Condensed tannin contents were expressed as mg catechin equivalent (CE)/g FGPE DW. All stock solutions were prepared in water/methanol (1:1, *v*/*v*). The ethyl acetate and n-hexane FGPE were initially dissolved in absolute DMSO at 200 mg/mL and subsequently diluted with water/methanol (1:1, *v*/*v*) to obtain 1 mg/mL stock solution. The water and 80% ethanol FGPE were directly prepared in water/methanol (1:1, *v*/*v*) at 1 mg/mL. All working dilutions were prepared from a stock solution of 1 mg/mL in water/methanol (1:1, *v*/*v*). The standards were prepared under the same condition as the samples.

### 4.4. Analysis of Phenolic Compounds by RP-HPLC

RP-HPLC system was used to analyze the phenolic compounds. The chromatographic conditions for the analysis of phenolic compounds were followed by Kubola & Siriamornpun (2011) with some modifications [18]. The analytical column was SuperSpher1 100, RP-18 cartridge (4.6 × 250 mm i.d., 5 µM) from Merck KGaA (Darmstadt, Germany). The flow rate was 0.8 mL/min, column temperature 38 °C, and diode array detected at 320 nm. Overall spectra were recorded from 200 to 600 nm. The complete methodological detail of HPLC is referred to in the Supplementary Materials.

### 4.5. Cell Culture

HT-22, an immortalized mouse hippocampal cell line (Sigma-Aldrich #SCC129), was used for this study. The cells were grown in Dulbecco’s Modified Eagle Medium (DMEM) purchased from Gibco (Life Technologies Corporation, Grand Island, NY, USA) supplemented with 10% fetal bovine serum (FBS) and 100 µL/mL penicillin–streptomycin at 37 °C with 5% CO_2_.

### 4.6. Cell Viability Assay

HT-22 cells were seeded in a 96-well plate at 6000 cells/well for 24 h. The cells were treated with various concentrations of FGPE or memantine (1, 5, 10, 50, 100, 500, and 1000 µg/mL) or Aβ_1–42_ (1, 1.5, 2, 2.5, and 3 µM) for 24 h. The tetrazolium salt methyl-thiazol-tetrazolium (MTT) assay was performed to check the cell viability [63]. Cells were washed with PBS, and 100 µL of MTT solution (0.5 mg/mL in PBS) was added to each well and incubated at 37 °C for 3 h. After that, MTT was removed and replaced with 100 µL/well of absolute dimethyl sulfoxide (DMSO) for 15 min with continuous stirring to dissolve formazan crystal as a final product of the MTT assay [64]. The absorbance was measured at 570 nm using BioTek SYNERGY H1 microplate reader (Winooski, VT, USA). FGPE, memantine, and Aβ_1–42_ concentrations were prepared in DMEM containing DMSO ≤ 0.05% (*v*/*v*). The results were expressed as a percentage of control (untreated cell) maintained in DMEM containing DMSO ≤ 0.05% (*v*/*v*).

### 4.7. Neuroprotective Assay

HT-22 cells were seeded in 96-well plates at 6000 cells/well for 24 h. Cells were exposed to 2.5 µM Aβ alone and with non-toxic concentrations of FGPE and memantine, respectively, for 24 h. The cells that were not subjected to any treatment and treated with Aβ alone were considered as sample control and Aβ control, respectively. The cell viability % was measured by MTT assay, and the morphology was observed using the light microscope (10× magnification).

### 4.8. Intracellular Reactive Oxygen Species (ROS) Detection by DHE Staining

Intracellular ROS levels were measured by using dihydroethidium (DHE) staining. DHE is a cell-permeable fluorogenic probe that reacts with superoxides to form ethidium and emits red fluorescence [65]. After treatment, the cells were washed with PBS and incubated with 10 µM DHE for 20 min. Then, cells were washed twice with PBS to remove the DHE stain. Representative fluorescence images were obtained by CellSens Olympus imaging software 4.1 using an inverted fluorescence microscope (Olympus, CKX53, Tokyo, Japan). All images were captured with the same fluorescent condition and exposure time. The intensity of red fluorescence was measured by using Image J software (version 1.54d; National Institutes of Health, USA) and three random non-adjacent fields in the group were used for statistical analysis.

### 4.9. Analysis of Mitochondrial Membrane Potential (MMP) by JC-1 Staining

The fluorescence probe JC-1 was used to detect MMP. After the treatment, the HT-22 cells were washed with PBS and incubated with 1 µM JC-1 dye (prepared in 5% FBS DMEM) at 37 °C for 30 min. At the end of incubation, cells were washed with PBS to remove the staining medium. Representative fluorescence images were obtained by CellSens Olympus imaging software 4.1 using an inverted fluorescence microscope (Olympus, CKX53, Tokyo, Japan), and results were represented by the ratio of the average red/green fluorescence intensity. Image J software (version 1.54d; National Institutes of Health, USA) was used to measure red and green fluorescence, and three random non-adjacent fields in the group were used for statistical analysis.

### 4.10. RNA Isolation and Real-Time Quantitative Polymerase Chain Reaction (RT-qPCR)

After treatment, total mRNA was isolated from the HT-22 cells using PureLink^TM^ RNA Mini Kit (Invitrogen, Carlsbad, CA, USA) following the supplier’s protocol. mRNA was reverse transcribed into cDNA by using a high-capacity cDNA reverse transcription kit (Applied Biosystems, Foster City, CA, USA). The cDNA templates were amplified using PowerUpTM SYBR Green Master qPCR Mix (Applied Biosystems) with specific primers of F 5′-AGATGAGGCTCACCGTCAACCT-3′ and R 5′-CCTCACCGTGTGCCATTCATTG-3′ for neurexin 2; F 5′ CAACGATGCTCTCCACAGGAGT-3′ and R 5′-CAGGTGAAACCTTCCCACTGCT-3′ for neurexin 3; F 5′-CGATGTCATGCTCAGCGCAGTA-3′ and R 5′-CCACACTACCTCTTCAAAGCGG-3′ for neuroligin 2; and F 5′-TCAGACGGTCACGATCATCGCT-3′ and R 5′-GTTGCTTCGCAGAGATGCAGTC-3′ for PSD-95. All mRNA levels were normalized to glyceraldehyde 3-phosphate dehydrogenase (GAPDH) with the following primers F 5′-CATCACTGCCACCCAGAAGACTG-3′ and R 5′-ATGCCAGTGAGCTTCCCGTTCAG-3′. Each measurement was tested in triplicate. Relative mRNA expression values were calculated with StepOne software (Applied Biosystems, version 2.3) using the cycle threshold (ΔΔct) method.

## 5. Statistical Analysis

All experiments were performed in at least three independent replicates. Data was presented as means ± standard deviation (±SD). Statistical significance was calculated by one-way ANOVA and independent pair t-test using SPSS statistic 17.0 software. Characters (*, **, and ***) and superscript letters (^a^, ^b^, and ^c^) indicated significant differences. The value of significant difference was considered at *p* < 0.05.

## 6. Conclusions

In conclusion, our study elucidated the multifaceted protective effects of FGPE, particularly the PE-EtOH and SE-EtOAc extracts, against Aβ-induced neurotoxicity in HT-22 cells. PE-EtOH and SE-EtOAc extracts significantly reduced ROS levels and improved MMP, thereby suggesting a potential therapeutic avenue. Further, the restoration of synaptic function was evidenced by the upregulation of PSD-95 expression, and maintaining neuroligin-2 suggested a nuanced mechanism by which these extracts enhanced synaptic plasticity and function. The protective effect of FGPE against Aβ-induced neurotoxicity not only contributed to our understanding of the underlying mechanism of neuroprotection and presented a promising avenue for the development of therapeutic strategies aimed at mitigating the effects of oxidative stress and mitochondrial and synaptic dysfunctions. Further research is needed to focus on elucidating the specific bioactive compounds within FGPE that confer neuroprotective effects in clinical settings.

## Figures and Tables

**Table 1:** Total polyphenol, flavonoid, and condensed tannin contents in FGPE.

FGPE	Phenolics (mg GAE/g EDW)	Flavonoids (mg QE/g EDW)	Condensed Tannins (mg CE/g EDW)
PE-H_2_O	15.85 ±1.11 ^e–h^	6.52 ± 0.63 ^e–g^	41.20 ± 4.43 ^e,f^
PE-EtOH	21.51 ± 2.05 ^d^	8.05 ± 0.76 ^e,f^	51.83 ± 3.68 ^e^
PE-EtOAc	47.81 ± 2.50 ^a^	16.09 ± 2.81 ^b,c^	44.15 ± 8.13 ^e,f^
PE-hexane	13.89 ± 3.72 ^f–i^	0.52 ± 0.08 ^i^	10.57 ± 2.24 ^g^
PU-H_2_O	10.95 ± 1.43 ^g–i^	8.36 ± 0.17 ^e,f^	40.70 ± 10.06 ^e,f^
PU-EtOH	12.29 ± 2.96 ^g–i^	12.84 ± 2.67 ^d^	117.54 ± 15.81 ^b^
PU-EtOAc	36.72 ± 3.05 ^b^	13.29 ± 0.34 ^c,d^	42.54 ± 5.29 ^e,f^
PU-hexane	11.22 ± 3.22 ^g–i^	5.78 ± 1.07 ^f,g^	13.59 ± 3.73 ^g^
AR-H_2_O	16.90 ± 0.47 ^e–h^	9.15 ± 0.27 ^e^	10.00 ± 7.40 ^g^
AR-EtOH	20.16 ± 0.10 ^d,e^	12.64 ± 1.93 ^d^	36.23 ±3.09 ^f^
AR-EtOAc	25.44 ± 3.29 ^c,d^	23.35 ± 1.08 ^a^	95.44 ± 12.56 ^c^
AR-hexane	10.06 ± 3.36 ^h,i^	4.12 ± 1.36 ^g,h^	33.66 ± 9.61 ^f^
SE-H_2_O	17.24 ± 1.30 ^e–g^	13.33 ± 1.97 ^c,d^	44.74 ± 3.09 ^e,f^
SE-EtOH	19.89 ± 0.65 ^d,e^	16.07 ± 0.88 ^b,c^	131.01 ± 6.81 ^a^
SE-EtOAc	31.03 ± 9.74 ^b,c^	16.85 ± 0.70 ^b^	90.85 ± 11.98 ^c^
SE-hexane	8.73 ± 2.95 ^i^	2.24 ± 0.22 ^h,i^	65.78 ± 5.02 ^d^

The data are expressed as mean ± standard deviation (SD) (n = 3). Differences among the 16 extracts for each chemical parameter (phenolics, flavonoids, and condensed tannins) were analyzed using one-way ANOVA followed by Tukey’s HSD test (*p* < 0.05). All pairwise comparisons were performed among the extracts within the same column. Means with different superscript letters (a–i) indicate significant differences. The letter “a” denotes the highest value, with subsequent lower values assigned consecutive letters of the alphabet, and “i” represents the lowest value. PE-H_2_O: water extract of peel; PE-EtOH: 80% ethanol extract of peel; PE-EtOAc: ethyl acetate extract of peel; PE-hexane: n-hexane extract of peel; PU-H_2_O: water extract of pulp; PU-EtOH: 80% ethanol extract of pulp; PU-EtOAc: ethyl acetate extract of pulp; PU-hexane: n-hexane extract of pulp; AR-H_2_O: water extract of aril; AR-EtOH: 80% ethanol extract of aril; AR-EtOAc: ethyl acetate extract of aril; AR-hexane: n-hexane extract of aril; SE-H_2_O: water extract of seed; SE-EtOH: 80% ethanol extract of seed; SE-EtOAc: ethyl acetate extract of seed; SE-hexane: n-hexane extract of seed; FGPE: fresh gac fruit parts extracts; GAE: gallic acid equivalent; QE: quercetin equivalent; CE: ±catechin equivalent; mg: milligram; g: gram; and EDW: extract dry weight.

**Table 2:** Phenolic acids and flavonoids content in PE-EtOH and SE-EtOAc.

	Gallic Acid	Chlorogenic Acid	Sinapic Acid	Rutin	Quercetin
PE-EtOH (µg/g EDW)	102.19 ± 11.19 ^b^	121.86 ± 24.32 ^b^	nd	1203.18 ± 15.68 ^a^	1.91 ± 0.13 ^c^
SE-EtOAc (µg/g EDW)	nd	nd	0.46 ± 0.09 ^b^	52.69 ± 10.74 ^a^	14.88 ± 7.46 ^b^

The data are expressed as mean ± standard deviation (SD) (*n* = 3). Differences in phenolic and flavonoid contents between PE-EtOH and SE-EtOAc were analyzed using one-way ANOVA followed by Tukey’s HSD test (*p* < 0.05). All pairwise comparisons were performed among the extracts within the same row. Means with different superscript letters (a, b, and c) indicate significant differences. The letter “a” denotes the highest value, with subsequent lower values assigned to consecutive letters of the alphabet, and “c” represents the lowest value. PE-EtOH: 80% ethanol extract of peel, SE-EtOAc: ethyl acetate extract of seed, nd: not detected, and EDW: extracts dry weight.

## Data Availability

The original contributions presented in this study are included in the article. Further inquiries can be directed to the corresponding author.

## Supplementary Materials

The following supporting information can be downloaded at: https://www.mdpi.com/article/10.3390/molecules30244767/s1, Figure S1: Cytotoxicity of FGPE at various concentration on HT-22 cells for 24 h. Cytotoxicity of (A) Peels extracts: PE-H_2_O, PE-EtOH, PE-EtOAc, PE-hexane (B) Pulp extracts: PU-H_2_O, PU-EtOH, PU-EtOAc, PU-hexane (C) Aril extracts: AR-H_2_O, AR-EtOH, AR-EtOAc, AR-hexane (D) seed extracts: SE-H_2_O, SE-EtOH, SE-EtOAc, SE-hexane. FGPE: fresh gac fruit parts extracts, PE-H_2_O: water extract of peel, PE-EtOH; 80% ethanol extract of peel, PE-EtOAc; ethyl acetate extract of peel, PE-hexane; n-hexane extract of peel, PU-H_2_O; water extract of pulp, PU-EtOH; 80% ethanol extract of pulp, PU-EtOAc; ethyl acetate extract of pulp, PU-hexane; n-hexane extract of pulp, AR-H_2_O; water extract of aril, AR-EtOH; 80% ethanol extract of aril, AR-EtOAc; ethyl acetate extract of aril, AR-hexane; n-hexane extract of aril, SE-H_2_O; water extract of seed, SE-EtOH; 80% ethanol extract of seed, SE-EtOAc; ethyl acetate extract of seed, SE-hexane; and n-hexane extract of seed. The data are expressed as mean ± standard deviation (SD) (*n* = 3). * *p* < 0.05, ** *p* < 0.01, *** *p* < 0.001 compared with control (0- untreated cell). Figure S2: Cytotoxicity of memantine at various concentrations on HT-22 cells for 24 h. The data are expressed as mean ± standard deviation (SD) (*n* = 3). * *p* < 0.05, ** *p* < 0.01 compared with control (0- untreated cell). Figure S3: Cytotoxicity of Aβ_1−42_ at various concentrations at various concentrations for 24 h. The data are expressed as mean ± standard deviation (SD) (*n* = 3). * *p* < 0.05, ** *p* < 0.01, *** *p* < 0.001 compared with control (0- untreated cell). Figure S4: Western blot analyses of Aβ_1−42_ incubated at 37 °C for different time intervals.
