# Supplementary material for: Neuroprotective Effect of Fresh Gac Fruit Parts Against β-Amyloid-Induced Toxicity and Its Influence on Synaptic Gene Expression in HT-22 Cell Model
Source: Molecules. 2025 Dec 13;30(24):4767. doi: 10.3390/molecules30244767 (PMC12735556; doi:10.3390/molecules30244767)
Supplement: Supplementary file 1 [file molecules-30-04767-s001.zip › Figure S1 Cytotoxicity of Fresh Gac Fruit Extracts.pdf]

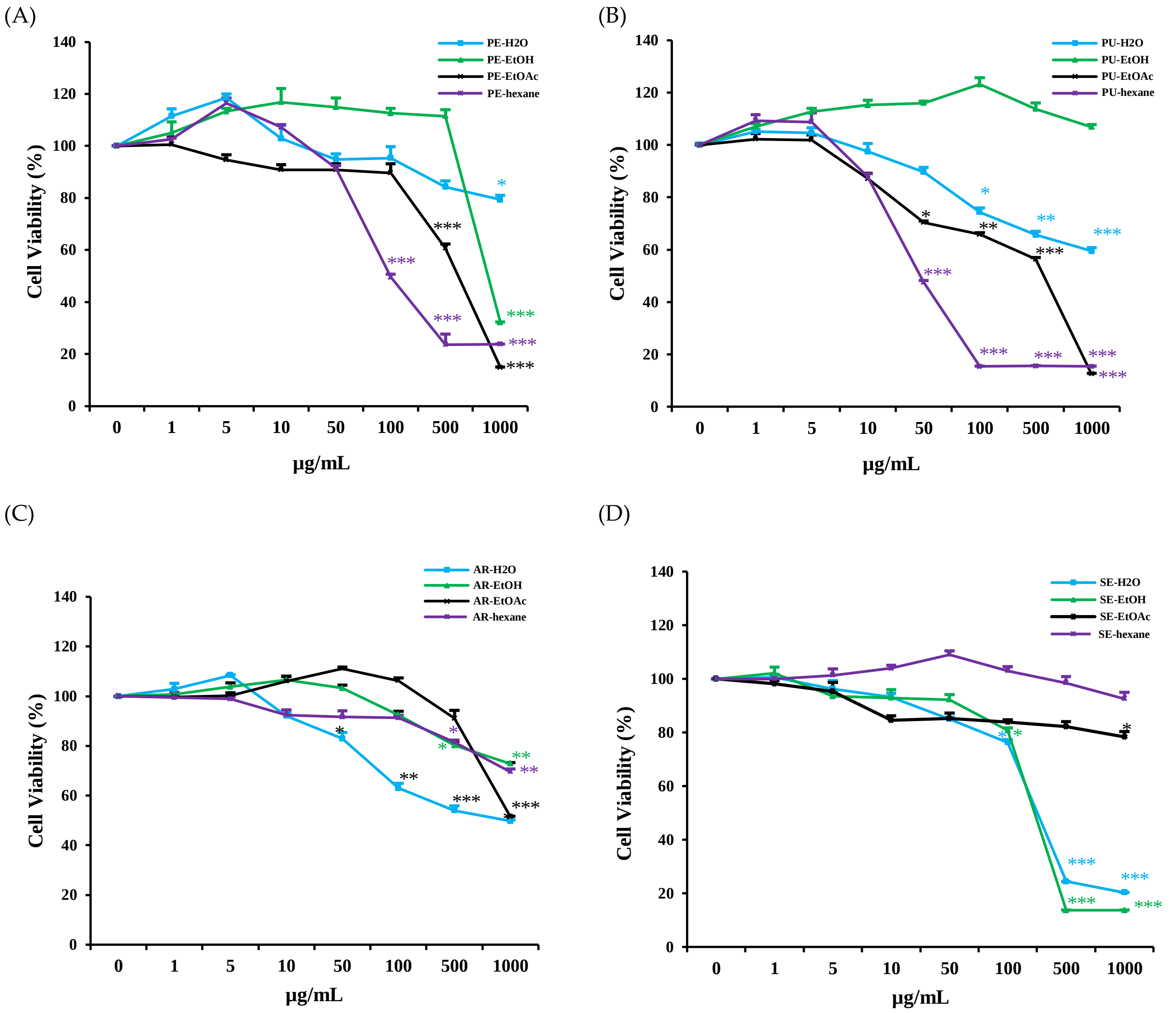

Figure S1: Cytotoxicity of FGPE at various concentration on HT-22 cells for 24 h. Cytotoxicity of (A) Peels extracts: PE-H_2O, PE-EtOH, PE-EtOAc, PE-hexane (B) Pulp extracts: PU-H_2O, PU-EtOH, PU-EtOAc, PU-hexane (C) Aril extracts: AR-H_2O, AR-EtOH, AR-EtOAc, AR-hexane (D) seed extracts: SE-H_2O, SE-EtOH, SE-EtOAc, SE-hexane. FGPE: fresh gac fruit parts extracts, PE-H_2O: water extract of peel, PE-EtOH; 80% ethanol extract of peel, PE-EtOAc; ethyl acetate extract of peel, PE-hexane; n-hexane extract of peel, PU-H_2O; water extract of pulp, PU-EtOH; 80% ethanol extract of pulp, PU-EtOAc; ethyl acetate extract of pulp, PU-hexane; n-hexane extract of pulp, AR-H_2O; water extract of aril, AR-EtOH; 80% ethanol extract of aril, AR-EtOAc; ethyl acetate extract of aril, AR-hexane; n-hexane extract of aril, SE-H_2O; water extract of seed, SE-EtOH; 80% ethanol extract of seed, SE-EtOAc; ethyl acetate extract of seed, SE-hexane; and n-hexane extract of seed. The data are expressed as mean ± standard deviation (SD) (n = 3). *$p < 0.05$, **$p < 0.01$, ***$p < 0.001$ compared with control (0- untreated cell).
