# Supplementary material for: Neuroprotective Effect of Fresh Gac Fruit Parts Against β-Amyloid-Induced Toxicity and Its Influence on Synaptic Gene Expression in HT-22 Cell Model
Source: Molecules. 2025 Dec 13;30(24):4767. doi: 10.3390/molecules30244767 (PMC12735556; doi:10.3390/molecules30244767)
Supplement: Supplementary file 1 [file molecules-30-04767-s001.zip › Figure S2 Cytotoxicity of Memantine.pdf]

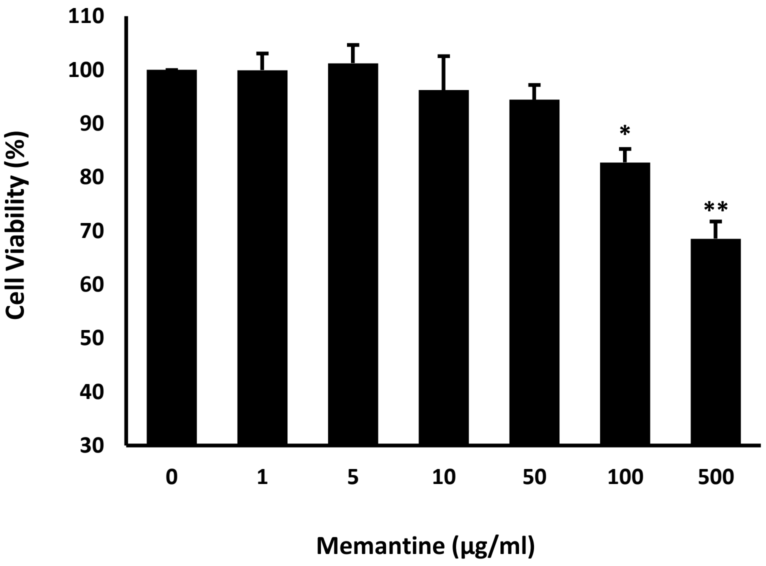

Figure S2: Cytotoxicity of memantine at various concentrations on HT-22 cells for 24 h. The data are expressed as mean ± standard deviation (SD) (n = 3). *p< 0.05, **p<0.01 compared with control (0- untreated cell).
