# Supplementary material for: Neuroprotective Effect of Fresh Gac Fruit Parts Against β-Amyloid-Induced Toxicity and Its Influence on Synaptic Gene Expression in HT-22 Cell Model
Source: Molecules. 2025 Dec 13;30(24):4767. doi: 10.3390/molecules30244767 (PMC12735556; doi:10.3390/molecules30244767)
Supplement: Supplementary file 1 [file molecules-30-04767-s001.zip › Figure S3 Cytotoxicity of Aβ1-42.pdf]

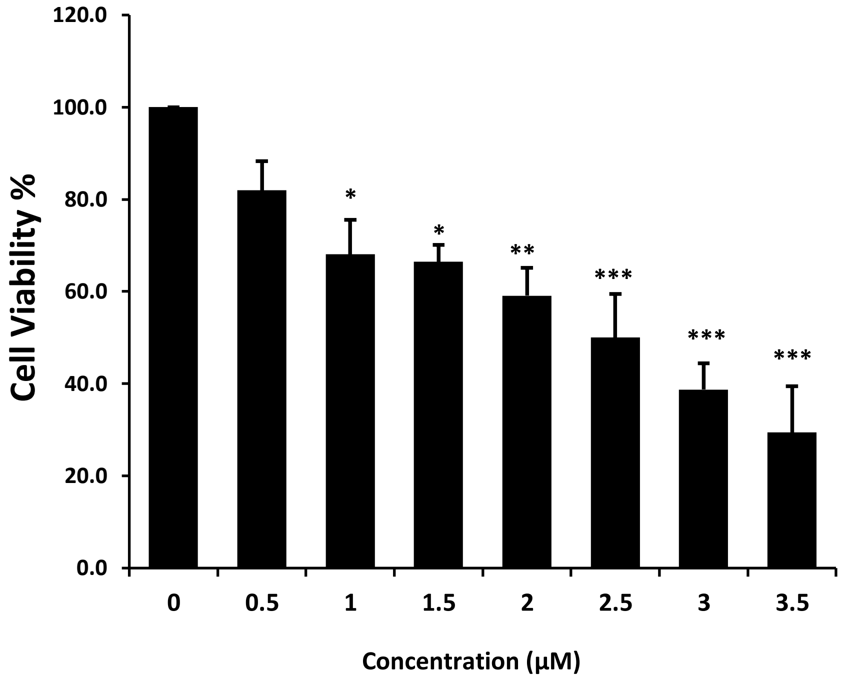

Figure S3: Cytotoxicity of A β 1-42 at various concentrations at various concentrations for 24 h The data are expressed as mean ± standard deviation (SD) (n = 3). *$p< 0.05$, **$p<0.01$, ***$p<0.001$ compared with control (0-untreated cell).
