# Supplementary figures and images for: Neuroprotective Effect of Fresh Gac Fruit Parts Against β-Amyloid-Induced Toxicity and Its Influence on Synaptic Gene Expression in HT-22 Cell Model
Source: Molecules. 2025 Dec 13;30(24):4767. doi: 10.3390/molecules30244767 (PMC12735556; doi:10.3390/molecules30244767)

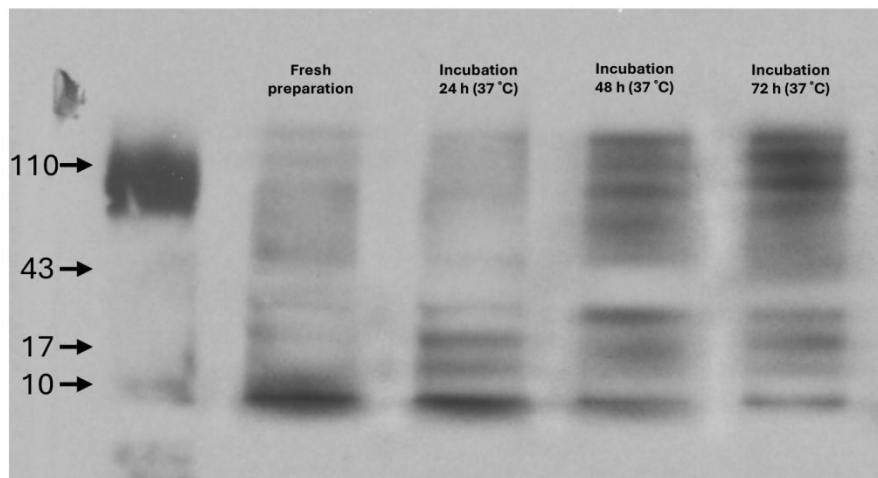

Figure S4: Western blot analyses of A $\beta$ <sub>1-42</sub> incubated at 37 °C for different time intervals.

Supplement: Supplementary file 1 [file molecules-30-04767-s001.zip › Figure S4 Western blot analyses of Aβ1–42 incubated at 37 °C for different time intervals.pdf]
